# Molecular identification of ligninolytic fungi from West Sumatra, Indonesia, using the Internal Transcribed Spacer (ITS) region

**DOI:** 10.5455/javar.2026.m1018

**Published:** 2026-03-10

**Authors:** Eli Ratni, Lendrawati Lendrawati, Muhammad Idris, Fadilla Hefzi

**Affiliations:** 1 Department of Animal Production Technology, Faculty of Animal Science, Universitas Andalas, Padang 25163, West Sumatra, Indonesia; 2 Department of Biology, Faculty of Mathematics and Natural Sciences, Universitas Andalas, Padang 25163, West Sumatra, Indonesia

**Keywords:** *Aspergillus*, ITS, ligninolytic fungi, ruminant feed, *Trichoderma*

## Abstract

**Objectives:**

This study aimed to isolate and identify ligninolytic fungi from decayed wood in West Sumatra, Indonesia, with a focus on their potential applications in animal production technology. The goal was to explore the potential of these fungi to improve lignocellulosic agricultural waste degradation, thereby enhancing the digestibility and nutritional value of ruminant feed.

**Materials and Methods:**

Fungi were isolated from rotting wood samples collected in Padang and Solok. The Bavendamm test was used to screen for ligninolytic activity. DNA was extracted from purified isolates, and PCR amplification of the ITS region was performed using ITS1 and ITS4 primers. Amplified products were sequenced and analyzed using BLAST against the NCBI GenBank database for species identification.

**Results:**

Two of four purified fungal isolates (P1 and S2) showed positive results in the Bavendamm test, indicating their ligninolytic potential. PCR amplification produced 574 bp (P1) fragments and 586 bp (S2). BLAST analysis identified P1 as *Trichoderma hamatum* and S2 as *Aspergillus flavus* with 100% similarity.

**Conclusions:**

Identifying *T. hamatum* and *A. flavus* as ligninolytic fungi from West Sumatra provides valuable insight for applying these species in animal production technology. These fungi could contribute to biodelignification processes, improving the utilization of lignocellulosic waste as a ruminant feed source.

## 1. Introduction

Animal feed ingredients are expected to increasingly compete with human food and raw materials for bioenergy production [1, 2]. Therefore, future livestock production systems should utilize underutilized organic matter and agricultural waste, such as straw, rice husks, bagasse, peanut shells, palm oil, and sugarcane, to maximize their potential. In tropical countries, where these agricultural by-products are abundant, there is a significant opportunity to incorporate them into animal feed. However, lignin, which provides rigidity to plant cell walls, is resistant to degradation under anaerobic conditions, such as those in the rumen [3, 4]. As a result, lignocellulosic biomass from tropical crops has limitations in supporting optimal rumen fermentation, thereby affecting digestibility, intake rate, and production performance in livestock. Feed ingredients should be selected for their high digestibility and reduced methane emissions [5], as well as their potential to utilize common tropical feed resources to enhance livestock productivity and mitigate environmental impacts.

Rumen microorganisms can treat lignocellulosic biomass in various ways to increase the accessibility of cellulose and hemicellulose, including physical, physicochemical, and chemical treatments. However, biological treatment is a more economical and environmentally friendly alternative [3]. One potential approach is the cultivation of fungal mycelium on the substrate, increasing the nutritional value of crop waste as ruminant feed. Fungi have a unique ability: they are the only organisms that can degrade the complex lignin structure of plant cell walls.

Fungal microorganisms capable of producing extracellular ligninolytic enzymes, such as laccase (Lac), manganese peroxidase (MnP), and lignin peroxidase (LiP), can degrade lignin [6, 7]. These enzymes have non-specific substrate preferences and play an important role in the breakdown of lignin compounds [8, 9]. Naturally, there are three groups of fungi known to degrade lignin: white-rot, brown-rot, and soft-rot fungi [9, 10]. This grouping is based on the weathering products: brown-rot fungi leave brown residues, while white-rot fungi leave white residues.

Fungal resources with ligninolytic activity are abundant in tropical countries, such as Indonesia, which have high species diversity. West Sumatra is one of the provinces rich in such resources. Therefore, identifying fungal species that can degrade lignin is an important step. Morphological characters typically guide the identification of fungal species. However, this approach often encounters obstacles, especially at the species level, because closely related species tend to exhibit high morphological similarity [11].

The development of molecular biology techniques enables the determination and identification of fungal isolates to the species level with greater accuracy [12, 13]. Polymerase chain reaction (PCR) techniques are typically used for molecular identification using markers such as the internal transcribed spacer (ITS) [14, 15, 16, 17]. ITS is a repetitive region of non-coding DNA sequences located in the coding region of ribosomal RNA molecules [15].

The knowledge gap in this field stems from the limitations of conventional morphological and biochemical identification methods, which often fail to distinguish closely related indigenous white-rot fungi. Although a preliminary study [18] identified several potential candidates, there is an urgent need for more precise species characterization to ensure the reliability of lignin-degrading applications. This research addresses this necessity by employing molecular identification based on the Internal Transcribed Spacer (ITS) region, widely recognized as the universal DNA barcode for fungi. Compared to other markers such as *18S rRNA*, the ITS region offers superior taxonomic resolution and greater interspecific variation, enabling more accurate identification of local isolates through comparison with extensive global databases. Consequently, the objective of this study was to isolate and identify indigenous ligninolytic fungal species from West Sumatra using ITS markers and to evaluate their potential for the biodelignification of lignocellulosic waste, ultimately enhancing the efficiency and sustainability of ruminant feed solutions.

## 2. Materials and Methods

### 2.1. Ethical approval

Ethical approval was not necessary for this study because it did not involve live animals, human participants, or any invasive procedures. All activities were conducted in compliance with applicable institutional and national guidelines for research and publication ethics.

### 2.2. Study area

We sampled two locations in West Sumatra: Padang (P), a low-altitude area, and Solok (S), a high-altitude area. Sample testing was conducted at Labor Biota Sumatra and Genetics Laboratory, Department of Biology, Faculty of Mathematics and Natural Sciences, Universitas Andalas, Padang.

### 2.3. Sample collection

Sampling was based on direct observation of rotting wood infected with fungi. We collected three different wood tissues from each location, so the total number of woods collected was six. We placed the collected samples into paper envelopes and labeled them P1, P2, P3, S1, S2, and S3. Further, we stored them at room temperature until the isolation process began.

### 2.4. Fungi isolation and purification

The isolation process commenced with the preparation of Potato Dextrose Agar (PDA) media. PDA powder (39 gm) was weighed and dissolved in 1000 ml of distilled water within an Erlenmeyer flask. The solution was heated and boiled on a hot plate stirrer until homogenized. Subsequently, the media were sterilized in an autoclave at 121°C for 15 min. Once the sterilized media reached a warm temperature, it was amended with chloramphenicol (Kemicetin) to prevent bacterial contamination. The media was then poured into Petri dishes under laminar airflow and allowed to solidify before the isolation procedure.

We took the infected wood parts, cut them into square shapes, washed them with distilled water, and then dried them using paper towels for the isolation process. We placed the dried wood pieces on PDA media, with three replicates, and incubated them for 3–4 days on a culture rack. Periodically, we observed the growth of the formed fungal colonies. We then purified the growing fungal colonies by transferring them to new PDA media. We kept the purified isolates as culture stock for further test preparation.

### 2.5. Primary screening test for ligninolytic activity

We conducted primary screening using the Bavendamm Test, which aims to identify the ligninolytic ability of fungi. We perform the Bavendamm test by adding 0.1% tannic acid to PDA media. We grew purified isolates on the media and incubated them for 7–10 days. We determine the test results based on the color change of the media. If no brown color is formed, the Bavendamm test is negative (-), indicating that the fungus is not ligninolytic. On the other hand, the formation of brown color yields a positive (+) Bavendamm test result, indicating that the fungus is ligninolytic.

### 2.6. DNA isolation and amplification

The fungal DNA isolation process used the Quick-DNA Magbead Plus Kit. The isolated DNA was then amplified using the PCR technique by mixing reaction components such as 12.5 µl MyTaq HS Red Mix Bioline (containing ten mM dNTPs, 50 m M MgCl^₂^, and 1-unit Taq DNA Polymerase), 3.5 µl nuclease-free water, 2 µl of ITS1 forward primer (5’-TCC GTA GGT GAA CCT GCG G-3’), 2 µl of ITS4 reverse primer (5’-TCC TCC GCT TAT TGA TAT GC-3’), and 5 µl of the DNA isolate.

We visualized the amplified DNA using electrophoresis on a 1% agarose gel. 2.5 µl of amplified DNA was inserted into each well of the agarose gel. We ran the electrophoresis at 100 V, 200 mA, and 20 W power for 55 min. After electrophoresis, the gel was placed on a UV transilluminator to document the amplification results.

### 2.7. DNA sequencing

We purified the PCR products and sent them to the 1st BASE Sequencing Services Laboratory (Selangor, Malaysia) for bidirectional sequencing.

### 2.8. Data analysis

The DNA sequencing results were contiguous when using the DNASTAR program. Furthermore, the DNA sequences were compared with sequences registered in public databases using the BLAST (Basic Local Alignment Search Tool), which can be accessed via the NCBI (National Center for Biotechnology Information) website at http://www.ncbi.nlm.nih.gov. The BLAST program was used to determine the similarity (similarity) level of the sequences with the data listed on the site. We then selected sequence data from the BLAST results in GenBank for alignment, phylogenetic tree construction, and genetic distance calculation using MEGA 11 [19].

## 3. Results and Discussion

### 3.1. Fungal isolates

**Figure 1.**
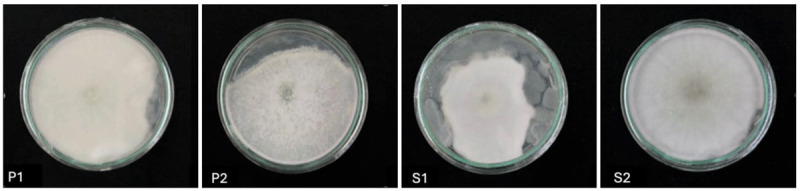
Ligninolytic fungi isolates (P = Padang, S = Solok).

Four fungal isolates that successfully grew on the medium were obtained from weathered wood in Padang and Solok, West Sumatra (Figure 1). Based on the observation results, the color of the isolated Colonie S is white, and there are hyphae. Fungi grow within their substrate, making their species diversity challenging to observe. Weathered or dead wood serves as the substrate for ligninolytic fungi. Researchers have documented more than 2500 species of fungi, including both basidiomycetes and ascomycetes, growing on dead wood [20, 21, 22].

### 3.2. Primary screening test for ligninolytic activity

**Figure 2.**
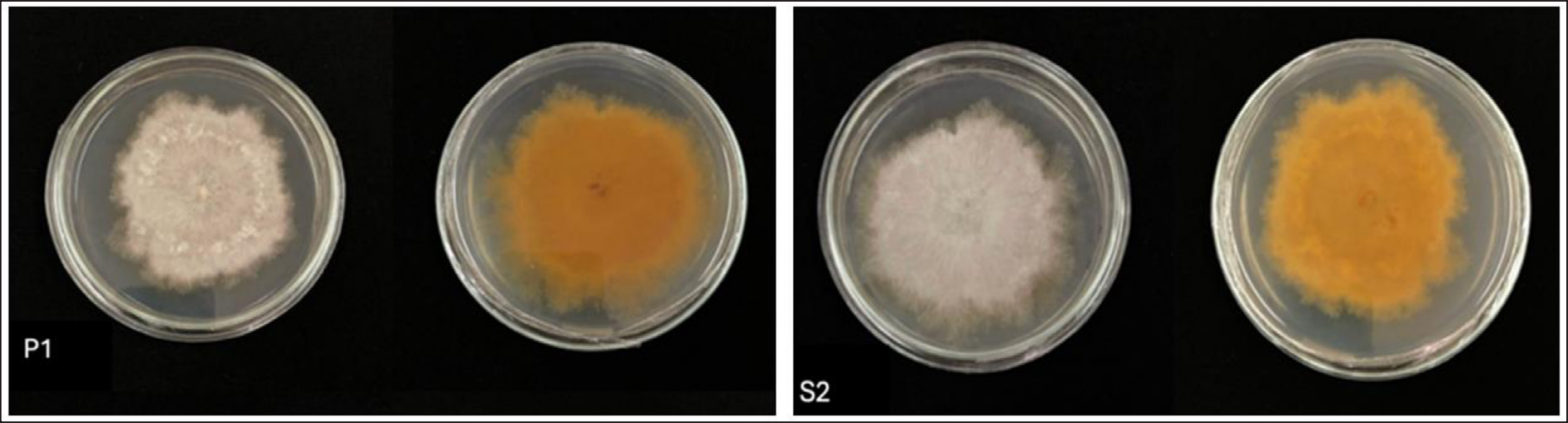
Bavendamm test results of fungi isolates that show positive results.

The results of the Bavendamm test on four purified isolates showed that two isolates, P1 and S2, were positive and characterized by the formation of a brown color in the media (Figure 2). This indicates that both isolates belong to the group of ligninolytic fungi.

The Bavendamm test is used to determine a fungus’s ability to produce extracellular phenol oxidase [23]. In addition, this test is also used to detect the activity and growth of extracellular polyphenol oxidase on lignocellulosic substrates [24].

### 3.3. DNA quantification

DNA isolation of ligninolytic fungi was successfully carried out using the Quick-DNA Magbead Plus Kit. After isolation, DNA quantification was performed using a Nanodrop spectrophotometer at 260 nm and 280 nm to determine the concentration and purity of the sample (Table 1).

**Table 1.** Concentration and purity of isolated ligninolytic fungi DNA.

No.	Isolate	Nucleic Acid Conc. (ng/µl)	A260/280	Volume (µl)
1	P1	121.9	2.02	50
2	S2	120.9	2.02	50

Based on Table 1, isolate P1 has a DNA concentration of 121.9 ng/μl with a purity of 2.02, while isolate S2 has a DNA concentration of 120.9 ng/μl with the same purity of 2.02. These values indicate that the DNA obtained from both isolates is of good quality, with high concentration and purity. Wood [25] defined good-quality DNA as having a concentration above 100 ng/l and a purity between 1.8-2.0. DNA purity values below 1.8 indicate the presence of contaminants, such as phenols or proteins, carried over during the isolation process, whereas values above 2.0 indicate possible contamination by RNA [26].

### 3.4. PCR amplification

**Figure 3.**
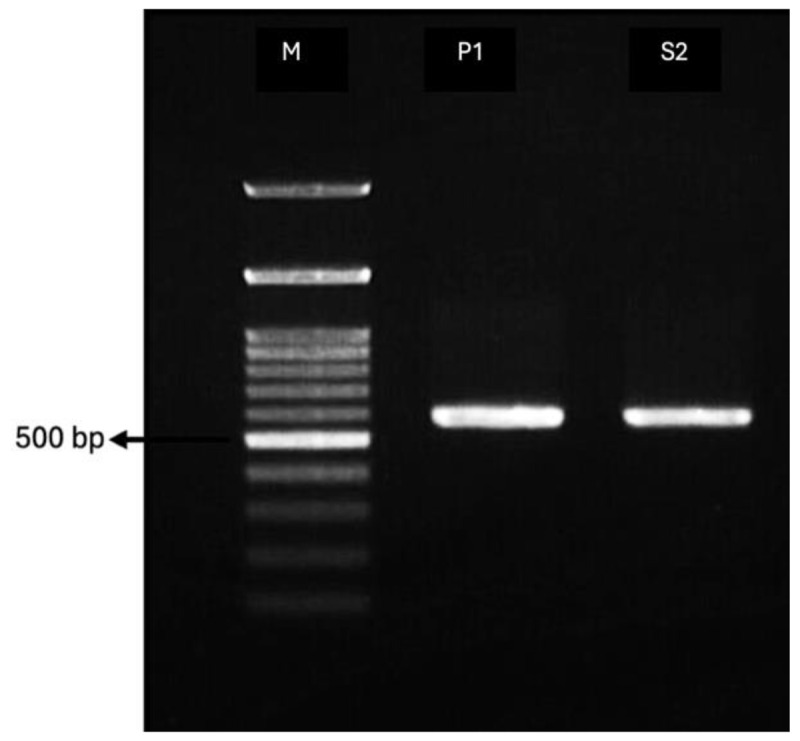
Visualization results of PCR products on 1% agarose gel (M = 100 bp DNA ladder).

DNA amplification of the two ligninolytic fungal isolates using ITS1 forward and ITS4 reverse primers produced products with fragment sizes of 574 bp for isolate P1 and 586 bp for isolate S2 (Figure 3). ITS1 and ITS4 are universal primers that amplify the ITS1 and ITS2 regions, including the 5.8S rDNA [27]. Fujita et al. [28] reported that amplification with ITS1 and ITS4 primers in fungi can yield amplicons ranging from 350 bp to 880 bp. Appiah et al. [29] also reported similar results, with fragment sizes of 400 bp for *P. ostreatus* and 600 bp for *S. commune* using ITS1 and ITS4 primers. Then, Apollos et al. [30] reported fragment sizes ranging from 450 to 650 bp in *Tricholoma robustum*. This difference in fragment size may be due to variations in the quality of DNA used in PCR [31, 32, 33]. Additionally, variability within the fungal ITS region, as well as differences in primer combinations used in amplification, can also affect the size of the resulting DNA bands [28, 34].

### 3.5. DNA sequencing

**Figure 4.**
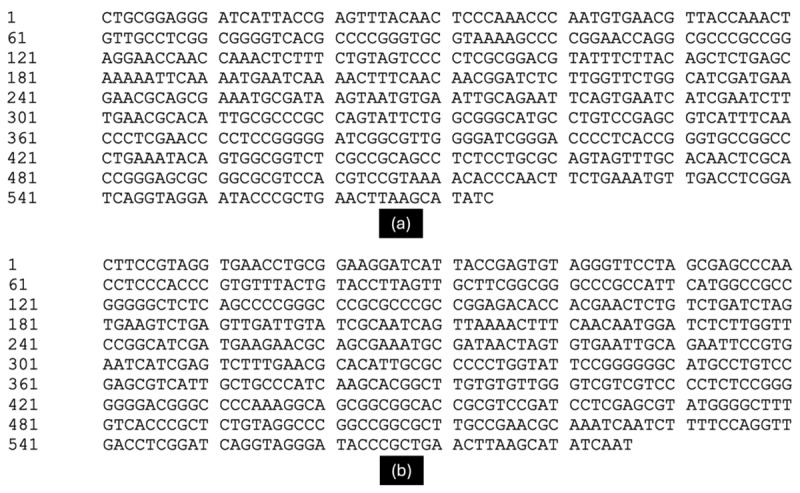
Complete ITS sequences of ligninolytic fungi isolates, (a) isolate P1, (b) isolate S2.

We purified and sequenced the amplification products using ITS primers to determine the nucleotide base sequence. The sequence of isolate P1 is 574 bp, while isolate S2 is 586 bp. Figure 4 displays the complete nucleotide base sequence of both isolates.

There was also a BLAST search of the complete ITS sequence of each isolate using the NCBI website (http://www.ncbi.nlm.nih.gov.BLAST) to find the homologous sequence of the sample DNA sequence with the nucleotide database in GenBank. This confirmed the similarity and allowed the type of ligninolytic fungus from the sample to be identified. Table 2 displays the top 10 BLAST results compared to the NCBI database.

Based on Table 2, the BLAST analysis shows that the isolated P1 is dominated by *Trichoderma hamatum*, with a similarity percentage of 100%. In contrast, isolate S2 is dominated by *Aspergillus flavus*, with 100% of the isolates. Therefore, we identified isolate P1 as *T. hamatum* and isolate S2 as *A. flavus*. Although *A. flavus* is commonly recognized as a potential aflatoxin producer, it was included in this study due to its robust ligninolytic enzyme system, which warrants further investigation regarding its safe application in bio-delignification processes. This decision is supported by Li et al. [9], who successfully isolated the *A. flavus* F-1 strain and demonstrated its superior ability to cleave lignin into smaller fragments, making it a promising candidate for transforming crop waste biomass. Consequently, these findings warrant further investigation into the enzymatic potential of isolate S2 while carefully considering its safe application in bio-delignification processes.

**Table 2.** BLAST search results showing the similarity.

	**P1 Isolate**					**S2 Isolate**				
**No**.	**Species**	**Query Cover**	**E-value**	**Percentage (%) Similarity**	**Accession**	**Species**	**Query Cover**	**E-value**	**Percentage (%) Similarity**	**Accession**
1	*Trichoderma hamatum*	100%	0.0	100%	ON927127.1	*Aspergillus flavus*	100%	0.0	100%	PP922360.1
2	*Trichoderma hamatum*	100%	0.0	100%	ON927099.1	*Aspergillus flavus*	100%	0.0	100%	MT529033.1
3	*Trichoderma hamatum*	100%	0.0	100%	OR435188.1	*Aspergillus flavus*	100%	0.0	100%	KX067887.1
4	*Trichoderma* sp.	100%	0.0	100%	OM760666.1	*Aspergillus flavus*	100%	0.0	100%	OQ422938.1
5	*Trichoderma* sp.	100%	0.0	100%	MW760777.1	*Aspergillus flavus*	100%	0.0	100%	PQ152254.1
6	*Trichoderma hamatum*	100%	0.0	100%	OR553890.1	*Aspergillus oryzae*	100%	0.0	100%	HQ285588.1
7	*Trichoderma* sp.	100%	0.0	100%	MK870109.1	*Aspergillus flavus*	100%	0.0	100%	MN095143.1
8	*Trichoderma hamatum*	100%	0.0	100%	DQ109530.1	*Aspergillus oryzae*	100%	0.0	100%	KY425742.1
9	*Trichoderma hamatum*	100%	0.0	100%	PP422096.1	*Aspergillus flavus*	100%	0.0	100%	MH590623.1
10	*Trichoderma hamatum*	100%	0.0	100%	MN264503.1	*Aspergillus flavus*	100%	0.0	100%	MK992254.2

From a taxonomic perspective, both *T. hamatum* and *A. flavus* are members of the phylum Ascomycota, although they diverge significantly at the ordinal and familial levels. *T. hamatum* is classified within the order Hypocreales and family *Hypocreaceae*, whereas *A. flavus* belongs to the order Eurotiales and family *Trichocomaceae*. Despite these phylogenetic differences, both genera are characterized by versatile metabolic pathways that enable them to thrive on lignin-rich substrates. Their presence in diverse ecological niches further highlights their capacity to secrete a complex array of extracellular enzymes essential for the degradation of recalcitrant lignocellulosic materials.

The functional divergence between the two isolates is further reflected in their enzymatic profiles. Members of the *Hypocreacea*e family, such as *Trichoderma*, are predominantly recognized for their robust cellulolytic activity, though they also contribute to lignin modification through laccase secretion [35]. Conversely, the *Trichocomaceae* family, particularly the *Aspergillus* genus, exhibits a more diverse array of lignin-modifying enzymes (LMEs), including lignin peroxidase (LiP) and manganese peroxidase (MnP), which are essential for the complete depolymerization of recalcitrant lignin [36]. This enzymatic synergy explains the high potential of both isolates for bio-delignification of agricultural waste, as they target distinct structural components of the plant cell wall.

While vigorous ligninolytic activity is traditionally associated with Basidiomycetes, certain Ascomycetes genera, including *Trichoderma* and *Aspergillus*, have demonstrated significant potential for lignin degradation through distinct enzymatic mechanisms [37, 38]. Unlike the aggressive delignification performed by Basidiomycetes, Ascomycetes often employ a ‘soft-rot’ strategy, which involves the gradual modification of the lignin-carbohydrate complex (LCC) via the secretion of laccases and auxiliary enzymes (AA9 family) [39, 40]. This approach is particularly effective in tropical environments, where these genera exhibit high adaptability and rapid colonization of lignocellulosic waste, making them efficient candidates for localized bio-delignification processes.

## 4. Conclusions

Based on the study results, two of the four purified ligninolytic fungal isolates, P1 and S2, showed positive results in the Bavendamm Test, indicating that both belong to the group of ligninolytic fungi. Molecular identification using the ITS region produced amplification products of 574 bp for isolate P1 and 586 bp for isolate S2. The BLAST analysis identified isolate P1 as *Trichoderma hamatum* and isolate S2 as *Aspergillus flavus*. These fungi could improve the degradation of lignocellulosic crop residues, especially in tropical countries, thereby enhancing the digestibility and nutritional value of ruminant feed. Importantly, their application may provide an economically beneficial strategy by enabling the utilization of locally available agricultural by-products as low-cost feed resources, reducing dependence on expensive commercial concentrates, and potentially lowering overall feeding costs for farmers.

## Data Availability

The data presented in this study are available from the corresponding author upon reasonable request.

## List of abbreviations

BLAST, basic local alignment search tool; DNA, deoxyribonucleic acid; ITS, internal transcribed spacer; Lac, laccase; LiP, lignin peroxidase; MnP, manganese-dependent peroxidase; MEGA, molecular evolutionary genetics analysis; NCBI, national center for biotechnology information; P, Padang city; PCR, polymerase chain reaction; PDA, potato dextrose agar; RNA, ribonucleic acid; S, Solok regency; sp., species.
